# Chronic Ingestion of H1-Antihistamines Increase Progression of Atherosclerosis in Apolipoprotein E-/- Mice

**DOI:** 10.1371/journal.pone.0102165

**Published:** 2014-07-14

**Authors:** Vineesh V. Raveendran, Donald D. Smith, Xiaoyu Tan, Matthew E. Sweeney, Gregory A. Reed, Colleen A. Flynn, Ossama W. Tawfik, Ginger Milne, Kottarappat N. Dileepan

**Affiliations:** 1 Division of Allergy, Clinical Immunology & Rheumatology, Department of Medicine, University of Kansas Medical Center, Kansas City, Kansas, United States of America; 2 Department of Pharmacology, Toxicology and Therapeutics, University of Kansas Medical Center, Kansas City, Kansas, United States of America; 3 Department of Pathology and Laboratory Medicine, University of Kansas Medical Center, Kansas City, Kansas, United States of America; 4 Vanderbilt University Medical Center, Nashville, Tennessee, United States of America; University Heart Center Freiburg, Germany

## Abstract

Although increased serum histamine levels and H1R expression in the plaque are seen in atherosclerosis, it is not known whether H1R activation is a causative factor in the development of the disease, or is a host defense response to atherogenic signals. In order to elucidate how pharmacological inhibition of histamine receptor 1 (H1R) signaling affects atherogenesis, we administered either cetirizine (1 and 4 mg/kg. b.w) or fexofenadine (10 and 40 mg/kg. b.w) to *ApoE^−/−^* mice maintained on a high fat diet for three months. Mice ingesting a low dose of cetirizine or fexofenadine had significantly higher plaque coverage in the aorta and cross-sectional lesion area at the aortic root. Surprisingly, the higher doses of cetirizine or fexofenadine did not enhance atherosclerotic lesion coverage over the controls. The low dose of fexofenadine, but not cetirizine, increased serum LDL cholesterol. Interestingly, the expression of iNOS and eNOS mRNA was increased in aortas of mice on high doses of cetirizine or fexofenadine. This may be a compensatory nitric oxide (NO)-mediated vasodilatory mechanism that accounts for the lack of increase in the progression of atherosclerosis. Although the administration of cetirizine did not alter blood pressure between the groups, there was a positive correlation between blood pressure and lesion/media ratio at the aortic root in mice receiving the low dose of cetirizine. However, this association was not observed in mice treated with the high dose of cetirizine or either doses of fexofenadine. The macrophages or T lymphocytes densities were not altered by low doses of H1-antihistamines, whereas, high doses decreased the number of macrophages but not T lymphocytes. The number of mast cells was decreased only in mice treated with low dose of fexofenadine. These results demonstrate that chronic ingestion of low therapeutic doses of cetirizine or fexofenadine enhance progression of atherosclerosis.

## Introduction

Histamine, a major product of mast cells, regulates many vital physiological functions including vasodilation, allergic response and neurotransmission [1]–[3]. The effects of histamine are mediated through a family of four G-protein coupled receptors (GPCR), histamine H1 receptor (H1R), H2R, H3R and H4R [4]. The role of histamine and H1R in the pathophysiology of atherosclerosis has been studied, with special emphasis on its potential proatherogenic role [5], [6]. In this regard, increased expression of H1R in human atheromas and elevated histamine content in atherosclerotic plaques in *ApoE* ^−/−^ mice have been reported [6], [7]. Furthermore, an elevated serum level of histamine is found to be associated with hypertension [8], atherosclerosis [9], and diabetes [10]. We have previously shown that histamine stimulates the production of both proatherogenic cytokines, such as IL-6, IL-8, and anti-atherogenic prostanoids, PGI_2_ and PGE_2_, in human coronary artery endothelial cells (HCAEC) through H1R activation [11]–[14]. Although, histamine and H1R signaling are associated with cardiovascular events, it is not known whether H1R activation is proatherogenic or is a protective immune response to atherogenesis. However, previous reports show that first generation H1-antihistamines like chlorpheniramine [15], and mepyramine [16] reduce atherogenesis in pigs and *ApoE^−/−^* mice, respectively. It is noteworthy that, recent studies from our laboratory demonstrated that new generation H1-antihistamines, cetirizine and fexofenadine, increase high fat diet-induced hepatic steatosis in C57Bl/6 mice [17]. The objective of this study was to examine the effect of chronic ingestion of these H1-antihistamines on progression of atherosclerosis in *ApoE^−/−^* mice.

## Methods

### Animals and treatments

Eight week old male B6/129P2-Apoe^tm^1Unc/J mice (*ApoE^−/−^*), stock number 002052 (Jackson Laboratories, Bar Harbor, ME) were randomly assigned into ‘placebo’, ‘cetirizine low’ (1 mg/kg b.w.), ‘cetirizine high’ (4 mg/kg b.w.), fexofenadine low (10 mg/kg b.w.), or fexofenadine high (40 mg/kg b.w.) groups. All mice were individually housed and fed *ad libitum* a Western diet (Harlan Teklad, Madison, WI) for three months. The experiments evaluating the effect of low dose cetirizine were carried out three times along with untreated controls. Data of these three experiments were combined for certain parameters as indicated in the results section. All animals had free access to either plain water or water containing cetirizine (5 mg/L and 20 mg/L, Sigma-Aldrich, St. Louis, MO) or fexofenadine (50 mg/L and 200 mg/L) to achieve an approximate daily intake of 1 and 4 mg cetirizine/kg b.w., or 10 and 40 mg/kg b.w. fexofenadine, respectively, assuming mice ingest approximately 5 mL of water/day. Fexofenadine solution was prepared by grinding Allegra tablets (over-the-counter, 180 mg/tablets) and filtering through Whatman filter paper to remove insoluble contents. Concentrations of H1-antihistmines were equivalent to the therapeutic doses for adult humans adjusted for the mouse body surface area [18]. The investigation conformed to the Guide for the Care and Use of Laboratory Animals published by the US National Institutes of Health, and was approved by the Institutional Animal Care and Use Committee of the University of Kansas Medical Center.

### Collection of urine, blood, heart and aorta

Twenty four hour urine samples were collected by placing each mouse in a metabolic cage (Tecniplast, Rochester, NY). Blood samples were collected from the retro-orbital sinus under anesthesia at the time of necropsy. Mice were euthanized using isoflurane (Abbott, North Chicago, IL) inhalation followed by opening the thoracic cavity. The heart and aorta were collected and processed for cross-sectional analyses of plaque area in the aortic root, and for *en face* analyses, as described previously [19], [20].

### Assessment of atherosclerotic plaques

Histological sections of the root of the aorta were stained with hematoxylin/eosin (H&E). Light microscopy was performed to evaluate the atherosclerotic changes. Histological sections on the glass slides were scanned using the Aperio Scanscope System (Vista, CA) to create virtual slides for quantitative evaluation. The images were transferred to Adobe Photoshop CS4 and lesion area at aortic root was measured using the lasso and analysis tool and presented as µm^2^. The lesion area is normalized to the underlying media area.

Aortas were prepared *en face* to quantify area of lesion coverage as described previously [19]. Briefly, the entire aorta was isolated from the arch to the aortic-iliac bifurcation. After removing the adipose tissue, the aorta was placed in 10% neutral buffered formalin overnight. The aorta was then opened lengthwise, and pinned flat in a wax bottomed dissecting pan. The tissue was stained for 15 minutes with 0.5% Sudan IV solution in acetone and 70% ethanol (1:1), then decolorized using 80% ethanol, and washed gently with water for 45–60 minutes. Each *en face* preparation was digitally imaged and transferred to Adobe Photoshop CS4 for the quantification of lesion coverage. To facilitate the conversion of pixels into mm^2^, a 10 mm scale was placed next to each aorta during photography. Lesions were delineated by tracing their contours with the Lasso tool and analyzed for the area of each plaque. After measuring the lesion areas, the percentage area of lesion coverage was calculated based on the total area of the aorta.

### Immunofluorescence staining of H1R in aortic roots

H1R protein expression in the atheroma was assessed in histological sections by using anti-H1R antibody (Alomone Labs, Israel) followed by Alexafluor-488 staining for immunofluorescence detection. Nucleus was counterstained with DAPI. Images were captured and analyzed using Leica TCS SPE Confocal Microscope as well as a Nikon 80i fluorescent microscope. Integrated density of H1R fluorescence was measured using Adobe Photoshop CC, Adobe Corporation.

### Quantification of T lymphocytes, macrophages and mast cells, and expression of scavenger receptor CD36

T lymphocytes (CD3+), macrophages (Mac3+) and mast cells (CD117+ or toluidine blue+) were quantified after immunostaining with anti-CD3 (DAKO, Carpinteria, CA), anti-Mac3 and anti-CD117 (eBioscience, San Diego, CA), respectively. The expression of CD36 was quantified using anti-CD36 antibody (Abcam, Cambridge, MA). After completion of immunohistochemical staining, slides were placed on the ACIS automated imaging system (DAKO) for quantifying the tissue staining. The system consists of an automated microscope, a three-chip Sony progressive scan camera, a computer, and Windows NT 4.0 workstation software interface. Each slide was scanned by the robotic microscope. The ACIS system captures images from each slide, quantifies staining in selected regions, and presents a numerical score. It was used to quantify immunohistochemical staining of percent positive cells for CD3 [19] and staining intensity for Mac3. An average score for all selected areas was then calculated for each marker. Mast cells were identified as toluidine blue+ and CD117+ cells and enumerated in three to five high power fields. The intensity of CD36 staining in aortic atheromas was graded on a scale from zero to three.

### Messenger RNA expressions in the aorta

Total RNA was extracted from the aorta of four animals from each group using TRlzol Reagent applying Phase Lock Gel-Heavy protocol at the University of Kansas Medical Center's microarray facility. Total RNA was reverse transcribed into first-strand cDNA using a High-Capacity cDNA Reverse Transcription Kit following the manufacturer's procedure. Quantitative real-time RT-PCR was performed using the ABI7500 Real-Time PCR system (Applied Biosystems). The amplification reactions were performed in 25 µl total volume containing SYBR Green PCR Master Mix with respective primers and 5 µl of cDNA of each mouse. The primers were designed using Primer Express Software v3.0 (Applied Biosystems). The sequences of primers used are provided in Table S1. The mRNA expressions of genes of each sample were normalized with their mRNA expression of β-actin. After normalization, the fold change of mRNA of each gene relative to one sample from the control group was calculated by comparative *ΔΔC_T_* method.

### Blood pressure monitoring

To obtain a measure of vascular functions associated with the treatments, blood pressure, heart rate and blood flow were measured at the end of experiment using a non-invasive tail cuff method (CODA 6, Kent Scientific). After restraining the mouse in a plastic holder, a small occlusion cuff and a Volume Pressure Recording (VPR) cuff were applied to the base of the tail. Fifteen cycles of BP readings were taken while six animals were simultaneously kept on a warming platform in a quiet, low lighted and low sensory environment. Animals were acclimated to the experimental protocol for 3 sessions before actual blood pressure measurements were recorded. The BP parameters and aortic plaque occlusion ratios were analyzed for correlation.

### Serum chemistry, lipid profiles and cytokine array

Serum chemistry, lipid profile and cytokine array were analyzed at Veterinary Laboratory Resources, Overland Park, KS and IDEXX Preclinical Research Services, West Sacramento, CA.

### Prostaglandin I_2_ and thromboxane A_2_ metabolites in the urine

Urine samples were purified and the levels of 11-dehydro thromboxane B_2_ and 2, 3-dinor-6-keto prostaglandin F_1α_ were quantified by gas chromatography-mass spectrometry (GC-MS) at the Jason. D. Morrow Laboratory, Vanderbilt University Medical Center, Nashville, TN or by 11-dehydro thromboxane B_2_ and 6-keto prostaglandin F_1α_ EIA kits (Cayman Chemicals, MI). The values for the prostanoid metabolites were normalized to creatinine levels.

### Histamine measurements in urine

Urine samples were analyzed for histamine levels by ELISA (SPI Bio, France) according to manufacturers' protocol.

### Cetirizine and fexofenadine levels in the serum

Serum samples collected from 3 untreated control mice and 6 mice treated with either cetirizine or fexofenadine were processed by solid-phase extraction and analyzed by reverse-phase liquid chromatography-tandem mass spectrometry (LC-MS/MS) for quantification of cetirizine or fexofenadine. The analytical procedure was based on the method of Flynn *et al.* [21].

### Statistical analysis

One-way or Two-way ANOVA were adopted for statistical analyses, as applicable. Pearson correlation analysis was used to investigate the relationships between blood pressure variables to lesion occlusion ratio. Results are presented as mean±SEM and p<0.05 is considered as significant. GraphPad Software v5.04 (San Diego, CA) was used to perform all the statistical analyses.

## Results

### Chronic ingestion of low doses cetirizine and fexofenadine increase atherosclerosis

**Figure 1:**
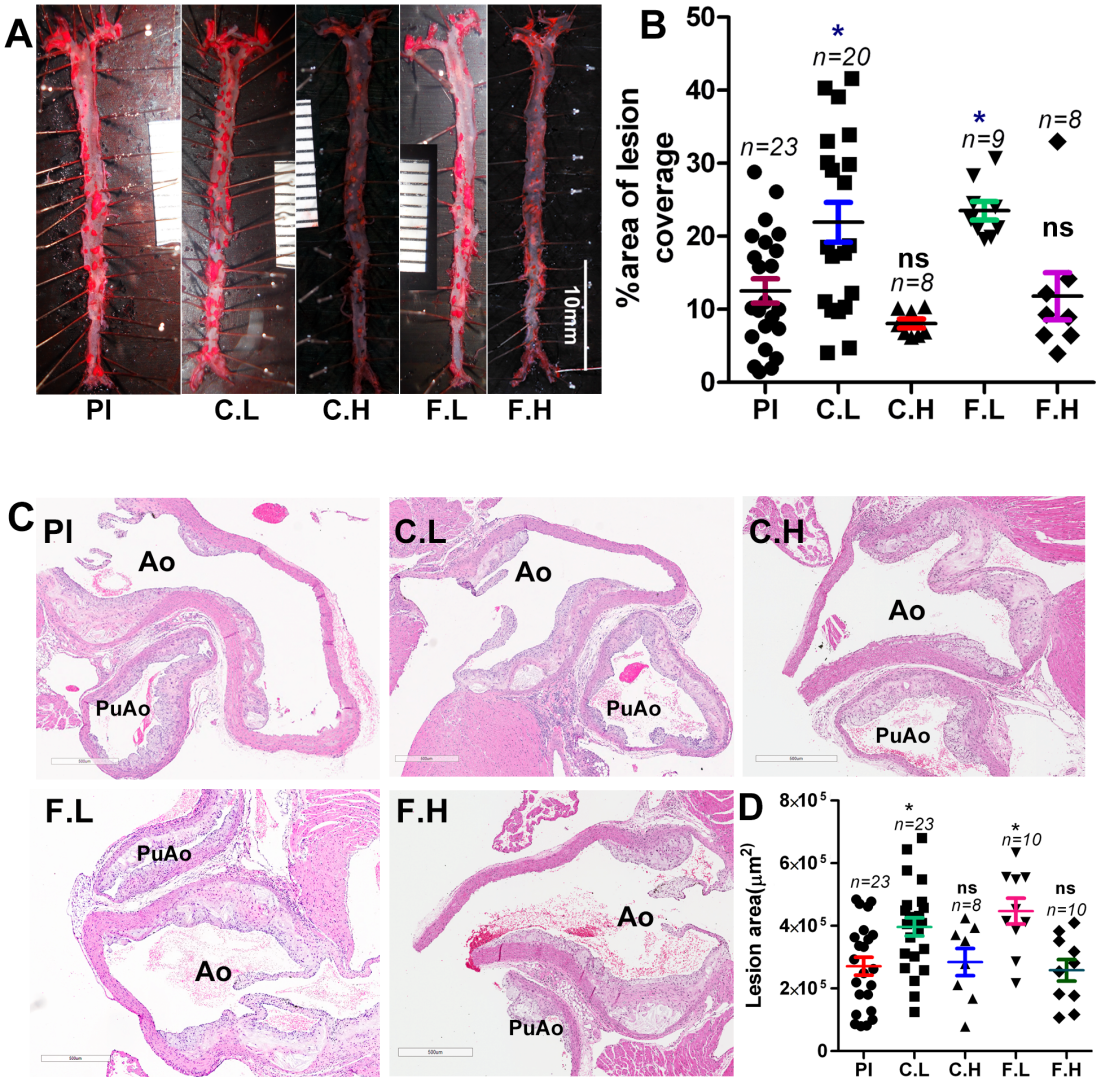
Effect of H1-antihistamines on atherosclerotic plaque development in the aorta. *ApoE^−/−^* ^mice^ maintained on a high fat diet were administered with cetirizine (1 and 4 mg/kg b.w) or fexofenadine (10 and 40 mg/kg b.w.) for 3 months in the drinking water. *ApoE^−/−^* mice receiving plain water served as placebo controls. Pl, placebo; C.L, cetirizine low; C.H, cetirizine high; F.L, fexofenadine low; F.H, fexofenadine high. Panel (A) represents images of *en face* preparations and (B) is the quantification of the percentage area of plaque coverage in the aorta. Representative images of the H & E stained cross-sections of the aortic root (C), and quantification of lesion area at the aortic root (D). Ao, aorta; PuAo, pulmonary artery. The “n” for Pl and C.L is the sum of mice used in three independent experiments. The “n” for C.H, F.L and F.H derived from a single experiment. Data points presented in B and D are mean ± SEM. *p<0.05 vs. control.

The results presented in Figure 1A and 1B demonstrate increased atheroma development in mice ingesting low doses of cetirizine or fexofenadine as measured by plaque coverage in the *en face* preparations. This proatherogenic effect of low doses of H1-antihistamines is further confirmed by the increased lesion area in the histological sections of aortic roots (Figure 1C and 1D). Interestingly, ingestion of the high doses of cetirizine or fexofenadine (Figure 1A–D) did not significantly enhance atherosclerosis over the placebo control.

### Cetirizine and fexofenadine do not increase H1R expression in the atheroma

**Figure 2:**
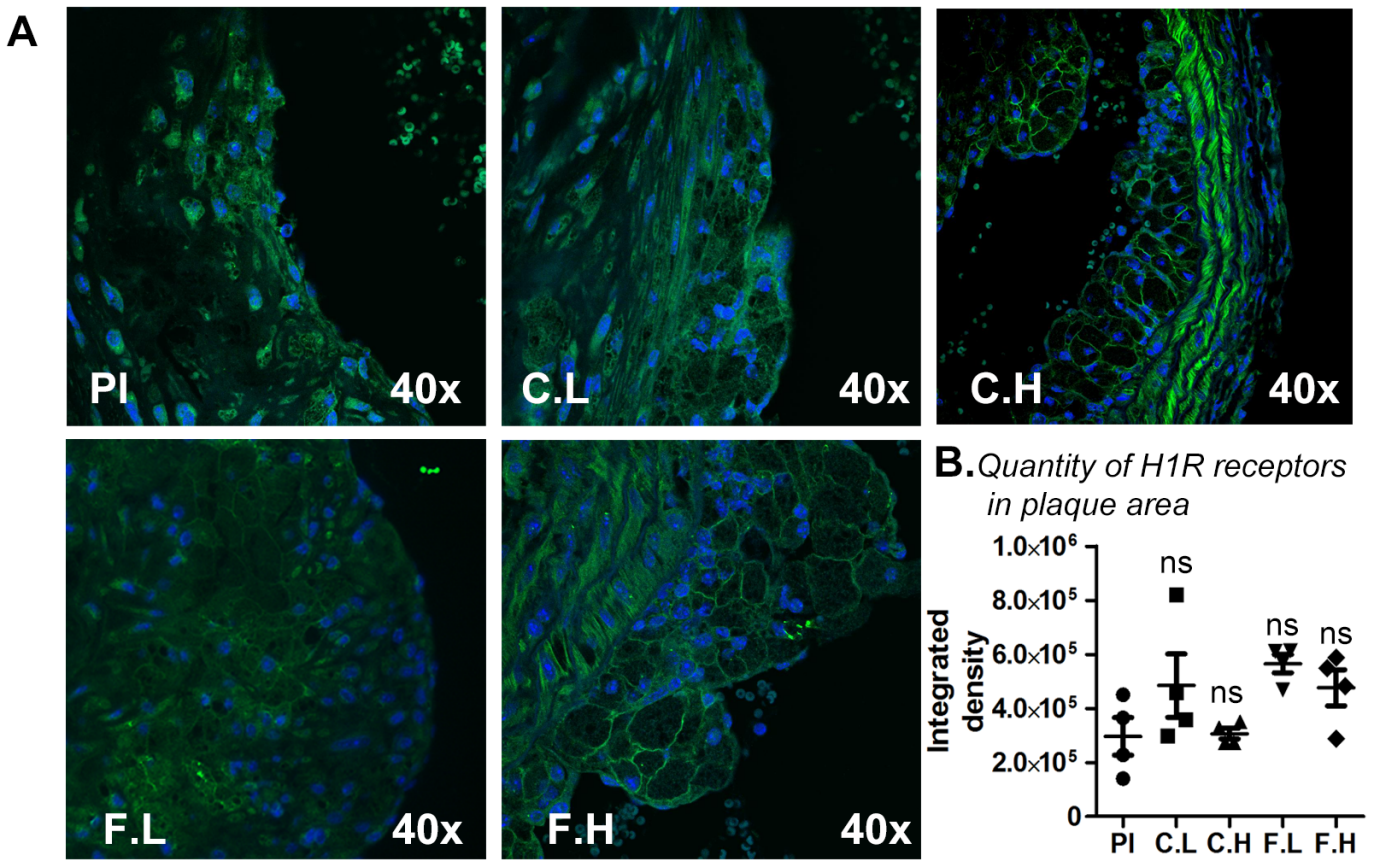
Effect of H1-antihistamines on H1R expression in atherosclerotic plaques. Atherosclerotic lesions around the aortic root of *ApoE^−/−^* mice were subjected to immunohistochemical analyses for H1R expression with anti-H1R (green) and DAPI (nuclear stain-blue). Panel (A) represents immunoflourescent staining for placebo (Pl), cetirizine low (C.L), cetirizine high (C.H), fexofenadine low (F.L) and fexofenadine high (F.H). Panel (B) depicts the integrated density of H1R staining. Magnification 40× at objective. n = 4.

Chronic ingestion of cetirizine or fexofenadine did not significantly alter H1R expression in the plaque area as determined by immunofluorescence analysis (Figure 2A and 2B).

### The increase in atherogenesis by low doses of H1-antihistamines is not associated with macrophage or T lymphocyte, but with a decrease in mast cell numbers in the lesions

**Figure 3:**
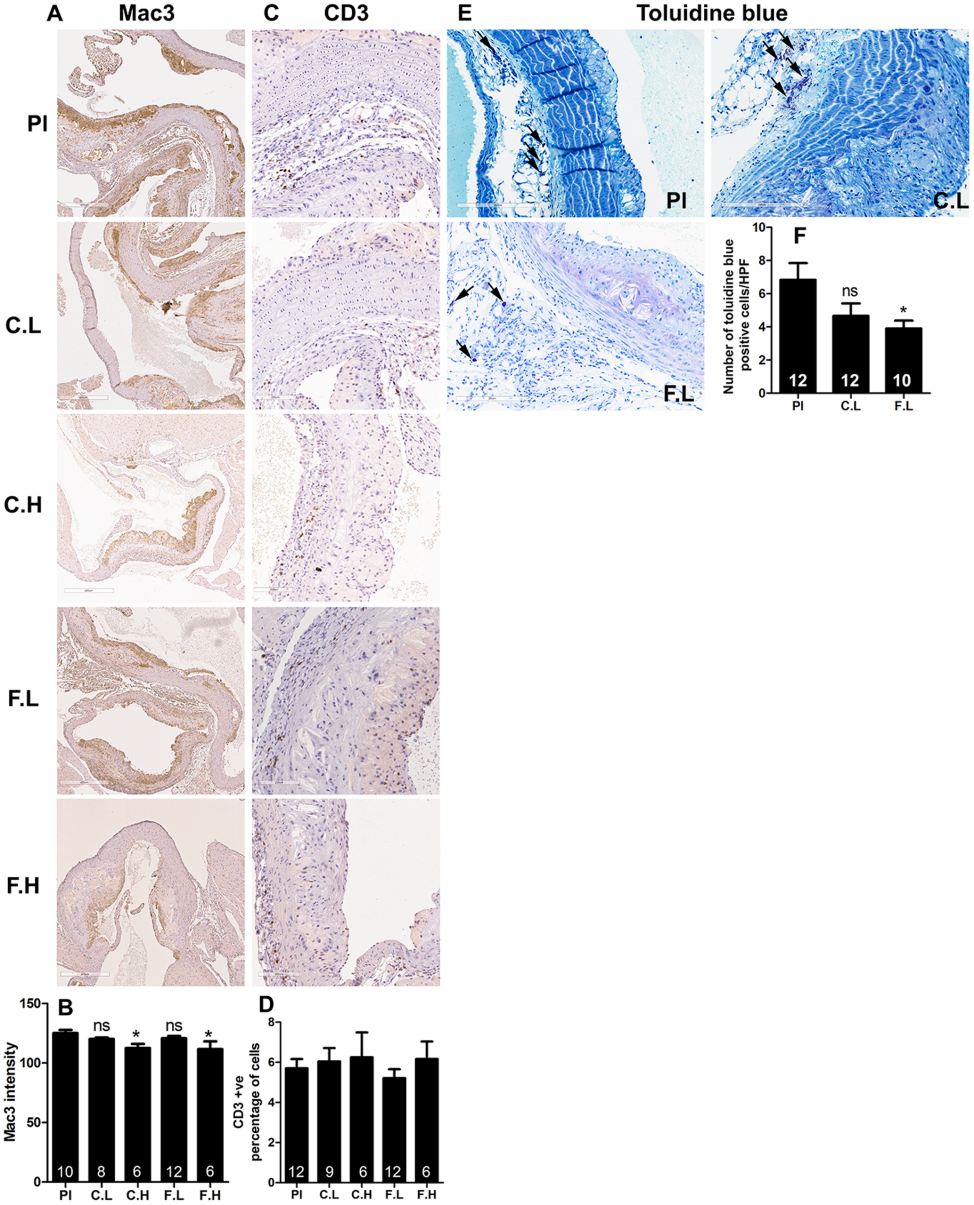
Effect of H1-antihistamines on recruitment of macrophages, T lymphocytes and mast cells into atheromas. Representative immunohistochemical images (magnification 200x) and quantitative data depicting the densities of Mac3 positive macrophages (A and B), CD3 positive T lymphocytes (C and D) and toluidine blue positive mast cells (D and E) are presented for placebo (Pl), cetirizine low (C.L), cetirizine high (C.H), fexofenadine low (F.L) and fexofenadine high (F.H). Results shown are mean ± SEM. Numbers of animals are indicated in each bar.

Increased numbers of T lymphocytes, macrophages and mast cells have been found to be associated with progression of atherosclerosis [22]–[24]. However, despite the increase in atherosclerosis in mice treated with the low doses of cetirizine or fexofenadine, we did not find increased number of macrophages (Figure 3A and 3B) or T lymphocytes (Figure 3C and 3D) in the lesions when compared to controls. On the other hand, the lack of increase in atheroma formation by high doses of cetirizine and fexofenadine was found to be associated with a decrease in macrophage population (Figure 3A and 3B). Although low doses of both H1-antihistasmines reduced mast cell numbers in atheroma, only the effect of fexofenadine was statistically significant (Figure 3E and 3F).

**Figure 4:**
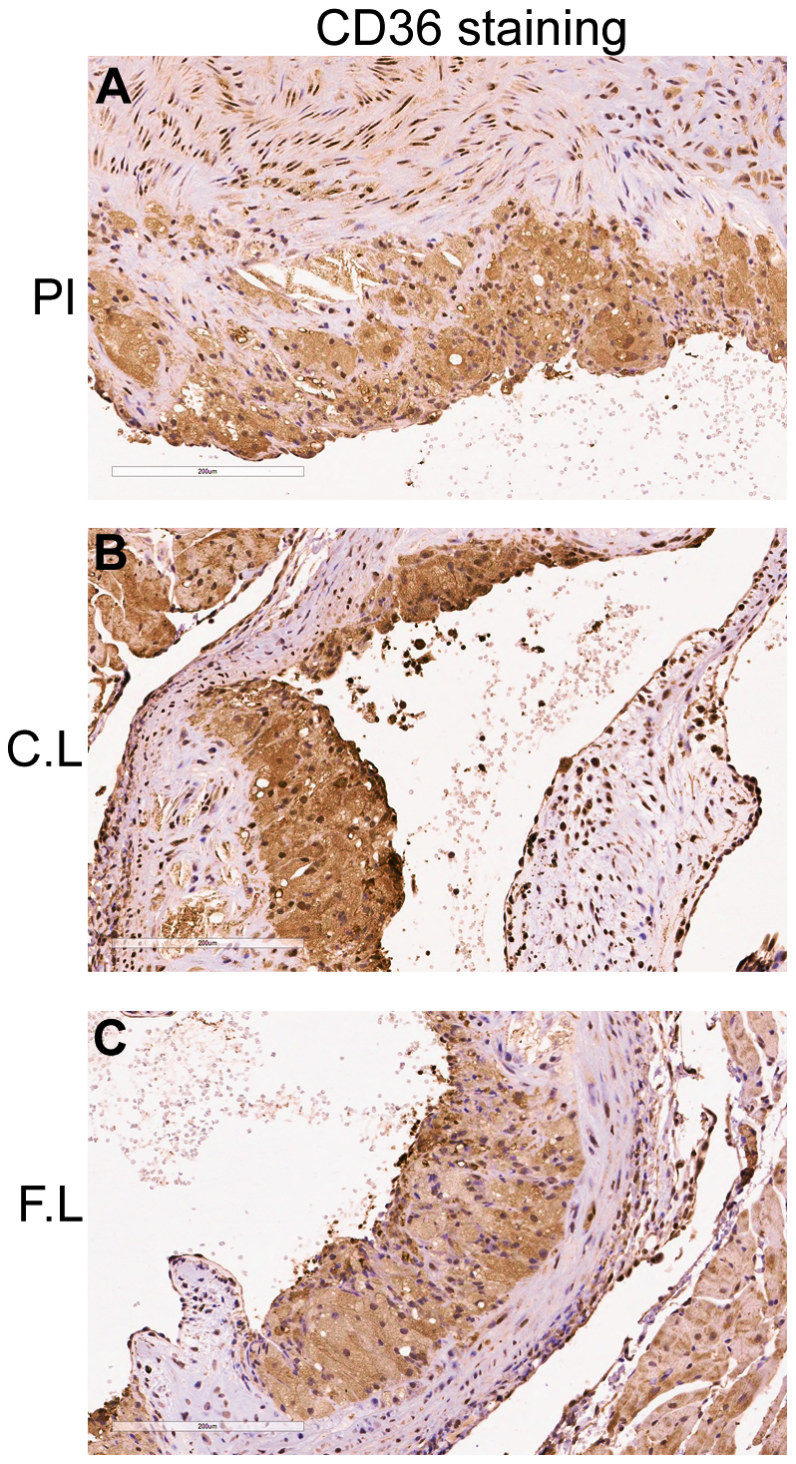
Effect of low doses of cetirizine and fexofenadine on CD36 expression. Representative immunohistochemical images (A–C, magnification at 200x) of scavenger receptor CD36 are presented. Placebo (Pl), cetirizine low (C.L), fexofenadine low (F.L). No significant difference in the intensity was noted between groups by a grading of scale zero to three.

Histological sections of the aortic root from 10–12 mice from placebo, low doses of cetirizine and fexofenadine groups were stained for scavenger receptor CD36 using specific antibody. The average scoring of the staining in aortic atheromas was similar in all groups of mice (Fig. 4).

### High doses of cetirizine and fexofenadine increases iNOS and eNOS mRNA expression in aorta

**Figure 5:**
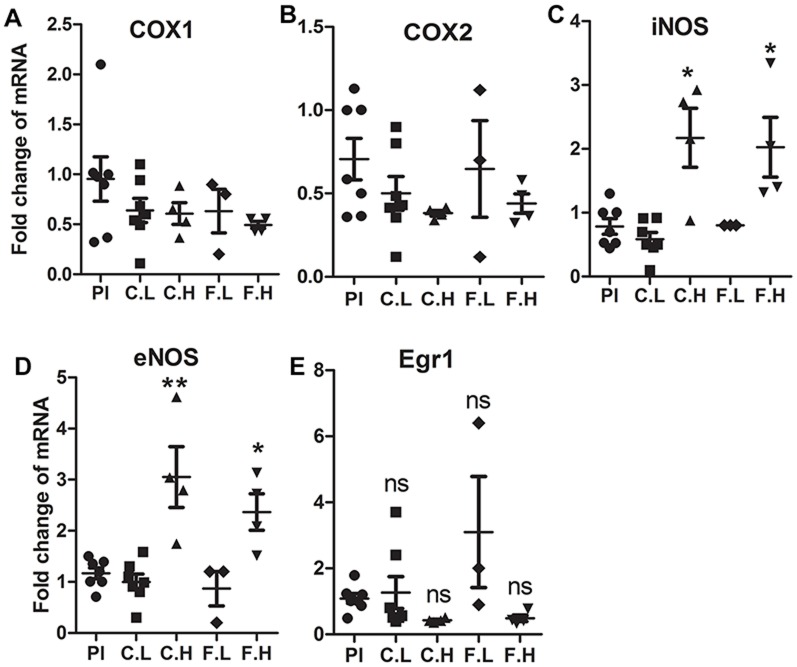
Effect of H1-antihistamines on the expression of COX1, COX2, iNOS, eNOS and Egr1 mRNA in aorta. Aortic tissues harvested from three or four mice from each group were analyzed for mRNA expression by qRT-PCR. Each gene amplicon was normalized for the β-actin expression. Placebo (Pl), cetirizine low (C.L), cetirizine high (C.H), fexofenadine low (F.L) and fexofenadine high (F.H). Data presented are mean ± SEM of the fold change compared to control values. *p<0.05 vs. controls.

The aortic tissues from mice treated with either the low or high doses of H1-antihistamines were analyzed for the expression of selected genes associated with vascular inflammation. The mRNA expression of COX isoforms were not altered by any of the doses of H1-antihistamines (Figure 5A and 5B). The expression of iNOS and eNOS mRNA expression were increased by high doses of both H1-antihistamines (Figure 5C and 5D). The expression of Egr1 mRNA was not significantly altered by any dose of cetirizine or fexofenadine (Figure 5E).

### Correlation of blood pressure with lesion/media ratio

**Figure 6:**
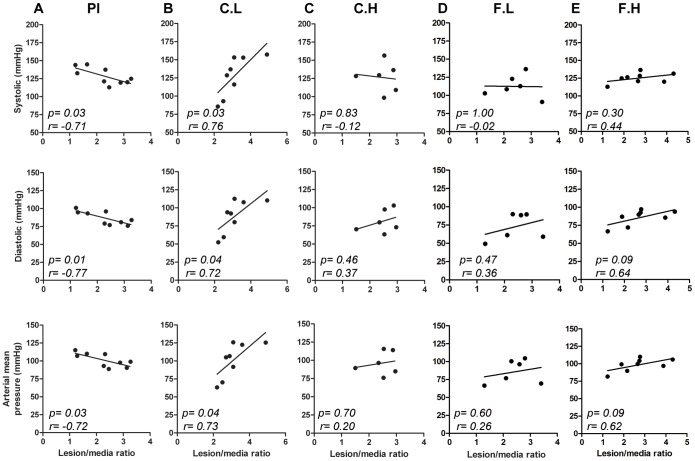
Correlation of blood pressure variables with plaque to media ratio. Correlation analyses between systolic, diastolic and arterial mean pressure values with the intima/media ratio of placebo, Pl (A), cetirizine low, C.L (B), cetirizine high, C.H (C), fexofenadine low, F.L (D) and fexofenadine high, F.H (E). The “*p*” values and Pearson “r” values are given for each graph. “n” = 9 for panel A; “n” = 8 for panel B and E, and “n” = 6 for panel C and D.

The vital parameters were not significantly different between the groups (Table 1). However, an analysis of correlation demonstrated that in low dose cetirizine treated mice, systolic, diastolic and arterial mean pressures were positively correlated with the lesion/media ratio. This is in contrast with placebo which showed a negative correlation. In contrast neither high dose of cetirizine nor any doses of doses of fexofenadine showed a correlation between BP parameters and the degree of occlusion (Figure 6).

**Table 1:** Blood pressure, heart rate and blood flow rate of *ApoE^−/−^* mice treated with H1-antihistamines.

Treatment	Systolic (mm Hg)	Diastolic (mm Hg)	Arterial Mean (mm Hg)	Heart Rate (bpm)	Flow (ml/min)	Volume (ml)
Placebo (n = 10)	127±4	87±3	101±3	716±24	11±1	43±3
Cetirizine Low (n = 12)	126±7	86±6	99±6	752±17	13±1	48±3
Cetirizine High (n = 11)	128±5	86±5	99±5	776±28	10±1	45±5
Fexofenadine Low (n = 6)	122±6	83±8	96±7	828±54	12±1	47±6
Fexofenadine High (n = 8)	125±3	86±4	98±3	772±24	11±2	45±7

The blood pressure was monitored using non-invasive tail cuff method. Each value is mean ± SEM.

### Serum lipids, chemistry and cytokines

Results of chronic ingestion of cetirizine and fexofenadine on serum lipids and chemistry are presented in Table 2. The low dose of cetirizine did not affect serum lipid levels. On the other hand, the low dose of fexofenadine significantly increased LDL cholesterol. The levels of creatine phosphokinase (CPK) were significantly decreased in low doses of both H1-antihistamines. Alkaline phosphatase (ALP) was increased only in mice ingesting low dose of fexofenadine.

**Table 2:** Serum lipid and chemistry profile of *ApoE^−/−^* mice treated with H1-antihistamines.

Parameters	Placebo	Cetirizine Low	Cetirizine High	Fexofenadine Low	Fexofenadine High
Total cholesterol	1067 ± 82(13)	1071 ± 65(13)	964 ± 96(6)	987 ± 44(6)	781 ± 64(6)
Triglycerides	189 ± 20(16)	210 ± 23(16)	177 ± 16(6)	134 ± 16(6)	226 ± 23(6)
HDL Cholesterol	19 ± 2(18)	20 ± 3(18)	14 ± 2(6)	18 ± 1(6)	13 ± 2(6)
**LDL Cholesterol**	208 ± 7(7)	212 ± 10(6)	213 ± 10(4)	253 ± 8^*^(6)	188 ± 6(2)
Glucose	213 ± 17(13)	181 ± 12(14)	209 ± 35(2)	234 ± 31(6)	ND
**CPK**	2076 ± 330(13)	659 ± 123^**^(14)	ND	1117 ± 188^*^(6)	ND
**ALP**	65.8 ± 9(12)	55.3 ± 6(12)	ND	107 ± 21^*^(6)	ND
SGOT	281 ± 22(13)	212 ± 22(15)	362 ± 115(2)	242 ± 37(6)	ND
SGPT	132 ± 20(12)	116 ± 16(15)	107 ± 30(2)	201 ± 57(6)	ND

ND = not determined, Each value is mean **±** SEM *p<0.05 and **p<.005.

The serum levels of cytokines and chemokines evaluated using cytokine array are presented in Table 3. Significant changes were noted only in IL-1α, macrophage inflammatory protein-1α (MIP-1α), MIP-3 and growth regulated kinase-a protein (GRO-α) by either of the H1-antihistamines.

**Table 3:** Serum cytokines.

Cytokines	Placebo	Cetirizine Low	Fexofenadine Low
Eotaxin (pg/ml)	1194±186	834±50	1195±103
**IL-1α (pg/ml)**	**376±60**	**291±52**	197±40*
IL-1β (ng/ml)	6.4±.4	7.3±0.8	7.1±0.4
IL-18 (ng/ml)	22.3±1.3	25.5±1.6	23.5±1.4
M-CSF-1 (ng/ml)	13.0±0.7	12.8±0.5	11.7±0.2
**MIP-1α (ng/ml)**	**7.2±0.1**	**8.1±0.4**	8.2±0.2*
MIP-1β (pg/ml)	545±49	593±63	627±75
MIP-2 (pg/ml)	130±45	59±19	86±14
**MIP-3 (ng/ml)**	**4.3±0.2**	3.5±0.2*	**4.6±0.2**
MCP-1 (pg/ml)	115±18	105±22	166±24
MCP-3 (pg/ml)	125±17	99±13	139±7
MCP-5 (ng/ml)	9.0±06	12.0±2.5	8.8±1.7
SCF (pg/ml)	793±65	907±125	865±55
TIMP1 (ng/ml)	1.2±0.2	0.9±0.1	1.0±0.1
Thrombopoeitin (ng/ml)	78.0±5.7	78.2±8.1	76.0±4.4
VEGF-α (pg/ml)	277±11.6	317±39	327±12
EGF (pg/ml)	88.7±20.3	69.7±8.3	83.0±12.3
**GRO-α protein (ng/ml)**	**0.20±0.04**	0.09±0.01*	**0.14±0.01**

Serum cytokines were determined by cytokine array commercially at IDEXX Preclinical Research Services. Each value is mean **±** SEM *p<0.05.

### Serum levels of cetirizine and fexofenadine

Animals in all groups consumed similar amounts of water and food (Data not shown). To evaluate the bioavailability of orally administered cetirizine and fexofenadine, we analyzed their levels in serum samples by LC-MS/MS. All animals in the cetirizine-treated groups had detectable levels of cetirizine with mean concentration of 190±50 ng/ml (n = 8; 4 mice each from experiment 1 and 2) and 710±146 ng/ml (n = 4, experiment 2) for mice treated with low dose and high dose cetirizine, respectively. As expected, cetirizine concentrations in sera of control mice were below the detectable limits of the assay (<3 ng/ml). The fexofenadine in serum of mice treated with high dose of fexofenadine was 8.4±0.3 ng/ml (n = 4). The serum levels of fexofenadine in the experiment with low dose of fexofenadine were not determined.

### Effect of H1-antihistamines on PGI_2_, TxA_2_ and histamine

**Figure 7:**
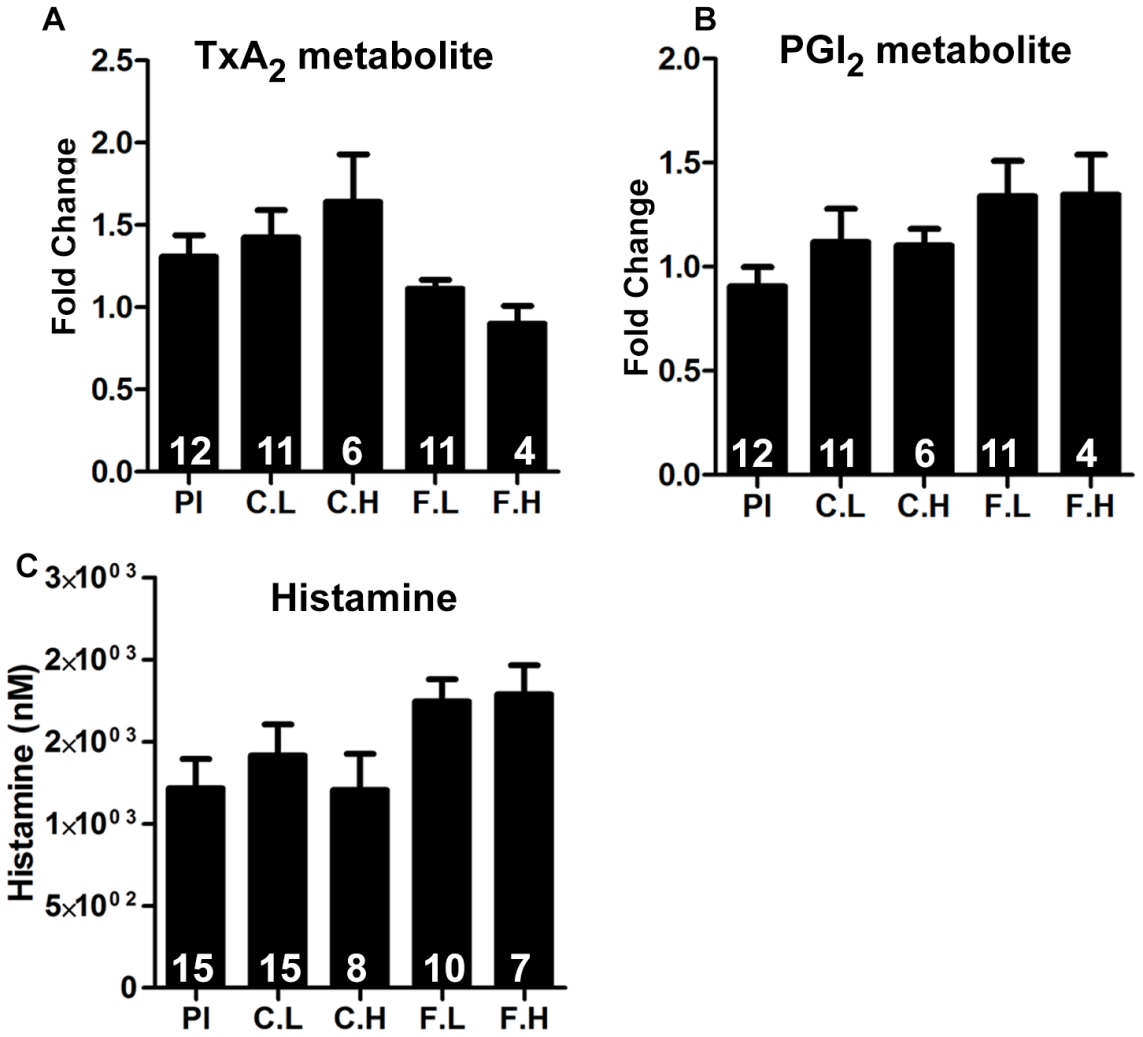
Effect of H1-antihistamines on levels of prostanoids and histamine. 24-h urine samples were analyzed for the concentration of stable metabolites of TxA_2_ (A), PGI_2_ (B) by GC-MS or EIA. Urinary levels of histamine were measured by EIA (C). Results shown are mean ± SEM. n =  4 to 12. None of the parameters found to be significant.

The cyclooxygenase pathway has an important influence in vascular inflammation and atherosclerosis. Of particular interest is the opposing effects of TxA_2_ and PGI_2_ on vascular tone and atherogenesis [25]. Histamine has been shown to enhance the expression of COX2 and the production of PGI_2_ in HCAEC via H1R activation [12]. To determine whether chronic ingestion of cetirizine and fexofenadine affect systemic production of TxA_2_ and PGI_2_, we measured the levels of the stable metabolites for TxA_2_ and PGI_2_ in the urine. Data presented in Figure 7A and B show that ingestion of H1-antihistamines does not affect systemic production of TxA_2_ or PGI_2_. Similarly, neither cetirizine nor fexofenadine affected urinary levels of histamine (Figure 7C).

## Discussion

Evidence suggests an important role for histamine and H1R in coronary artery disease and atherosclerosis [6], [7], [26]. A recent report showed that either the treatment with a first generation H1-antihistamine mepyramine or deletion of H1R gene in *ApoE^−/−^* mice resulted in significant reduction of atherosclerotic lesion formation [16]. In this present study, we show that oral administration of human therapeutic doses of cetirizine (1 mg/kg b.w) or fexofenadine (10 mg/kg b.w) in *ApoE^−/−^* mice for three months did not reduce progression of atherosclerosis, instead, increased the atherosclerotic lesion formation. These results are not in agreement with the antiatherogenic effect recently reported for mepyramine [16]. We recognize the fact that H1R gene deletion was able to mimic the effect of mepyramine in the earlier study. We believe that distinct pharmacological features of mepyramine and those of cetirizine and fexofenadine, and differences in the doses of H1-antihistamines used, might be the contributing factors for the disparity between the present and previous report [16].

Cetirizine and fexofenadine are extensively used for treating allergic diseases. To the best of our knowledge, this is the first study which compared the effects of therapeutic doses of two commonly used H1-antihistamines on the progression of atherosclerosis in the *ApoE^−/−^* mouse model. In this study, we demonstrate that lesion formation in the entire aorta was exacerbated by low dose H1-antihistamines. In addition, low doses of these H1-antihistamines increased lesion thickness and area at the aortic root. To our surprise, high doses of cetirizine or fexofenadine did not enhance atheroma formation compared to controls. This can be explained by the unique properties of H1R inverse agonists, which depends on the affinity and the concentration of drug [27]. The lower doses of H1-antihistamines may not be sufficient to completely block H1R signaling especially if H1 receptors are over expressed. This contention is supported by the finding that low concentrations of both cetirizine and fexofenadine seem to enhance H1R expression compared to control as well as their respective high dose counterparts (Figure 2A). In the case of high doses of H1-antihistamines, more efficient blockade of H1R signaling should be expected. It is possible that further increases in the doses of these antihistamines may even reduce atherogenesis. This may explain the discrepancy between our findings and the previous report demonstrating reduced atherogenesis by mepyramine or H1R deletion in *ApoE^−/−^* mice. Also, unique chemical and pharmacological properties of these different H1-antihistamines secondary to antagonizing H1R signaling may result in their adverse outcomes.

A second possibility is that high doses of these H1-antihistamines switch H1R into an inactive conformation because of their inverse agonistic properties. The molecular basis of the mode of action of second generation antihistamines demonstrates unique receptor binding characteristics, since they bind to H1R as inverse agonists rather than antagonists [28]. Since cetirizine and fexofenadine are inverse H1R agonists, they can generate opposite pharmacological effects compared to the agonist. Thus, in the absence of agonist binding, an inverse agonist can induce overwhelming negative impacts, especially under *in vivo* conditions with chronic blockade of the receptor signaling. It is possible that higher doses of inverse agonists may irreversibly modulate the receptor signaling or promiscuously bind to other histamine receptors such as H4R [29]. This may explain the results of increased atherogenic effects of cetirizine and fexofenadine at the low doses, and not at the high doses.

Atherogenesis involves increased recruitment of macrophages, T lymphocytes and mast cells in a lesion [30], [31]. Macrophages are critical in atherogenic initiation as they transform to foam cells. It has been reported that the T lymphocyte deficiency reduces lesion formation during moderate hypercholesterolemia [32]. Increased number of mast cells are known to be present in the adventitia of atherosclerotic lesions [31]. In the present study, the increase in lesion formation by low doses of cetirizine or fexofenadine was not associated with an increased macrophage or T lymphocyte count. High doses of these H1-antihistamines, on the other hand, were found to reduce the macrophage content without changing the T lymphocytes. Low dose of cetirizine or fexofenadine reduced mast cell recruitment into the plaque area although the effect of cetirizine was not statistically significant. This is opposite to the contention that mast cells promote atherogenesis [19]. This reduction in mast cell numbers in the plaque might be due to increased degranulation. The amount of scavenger receptor CD36 was also found not to be affected by low doses of H1-antihistamines.

Cytokines and chemokines derived from activated mast cells, macrophages and other cell types have unique effects in propagating proinflammatory and atherogenic signals. This study provides insight into the alterations in serum cytokine and chemokine levels in *ApoE^−/−^* mice treated with H1-antihistamines. In the present study, concentrations of various proatherogenic cytokines are altered by low dose of cetirizine or fexofenadine (Table 3). Proatherogenic cytokines such as IL-1α and MIP-3 levels are reduced in fexofenadine or cetirizine-treated mice, respectively. In contrast, MIP-1α was increased in fexofenadine treated mice. The levels of VEGF-α tended to increase in both cetirizine and fexofenadine-treated mice. Proinflammatory cytokines such as TNF-α, IL-6, IFN-γ were below detectable levels in the cytokine array. In this study, the cytokine array did not identify any particular cytokine or chemokine responsible for the proatherogenic outcome of low dose H1-antihistamines. Quantification of these putative cytokines using high sensitivity assays are needed for conclusive interpretations.

We noted significant increases in iNOS and eNOS mRNA expression in the aortic tissues of mice treated with high doses of cetirizine or fexofenadine. Although we did not measure the levels of nitric oxide in vasculature, we speculate that this may be nitric oxide-mediated compensatory mechanism to regain the vascular tone affected by H1R blocking. Furthermore, since nitric oxide is a powerful vasodilator and inhibitor of platelet aggregation, it may act as an antiatherogenic factor [33]. Thus, the relatively low level expression of iNOS, eNOS, COX1 and COX2 in animals treated with low dose cetirizine or fexofenadine suggests that these animals may experience persistent vasoconstriction compared to high dose H1-antihistamine-treated animals. In order to examine the potential association between cetirizine or fexofenadine-mediated changes in atheroma development and vascular functions, we measured the blood pressure and associated parameters. We did not find any difference in the blood pressure values between the groups. However, the systolic, diastolic and mean arterial pressures were positively correlated with lesion-media ratio at the aortic root in low dose cetirizine-treated mice. Surprisingly a negative correlation between blood pressure and lesion area was noted in control mice. This may be explained by the ongoing histamine/H1R mediated vasodilation in the absence of H1-antihistamines. In respect to mice treated with high dose cetirizine or fexofenadine, or low dose fexofenadine, there was no correlation between blood pressure and lesion-media ratio. This may be attributed to increased vasodilatory effects induced by increases in nitric oxide levels derived from overexpressed eNOS and/or iNOS.

Hypercholesterolemia is a characteristic feature of atherosclerosis in mouse models and humans, although the condition does not always correlate with the progression of the disease [20], [34], [35]. In this study, the serum levels of triglycerides, total cholesterol, LDL, HDL, and VLDL were not altered in the *ApoE^−/−^* mice treated with low dose cetirizine although these animals had increased atherosclerotic plaque area. On the other hand, low dose fexofenadine significantly increased LDL cholesterol levels. This suggests that lipid derangement may be a cause in fexofenadine, but not in cetirizine-induced increase in atherosclerosis.

In summary, the low therapeutic doses of cetirizine or fexofenadine increase atherosclerotic lesion formation in *ApoE^−/−^* mice maintained on a high fat diet for three months. Interestingly, *ApoE^−/−^* mice treated with high doses of cetirizine or fexofenadine did not increase plaque development when compared to controls. It should be noted that increased atherosclerosis by low dose cetirizine was not associated with lipid derangements. But low dose of fexofenadine showed an increase in LDL cholesterol levels which correlated with atherosclerosis. However, we noted a positive correlation between blood pressure and lesion area in low dose cetirizine- treated mice. The high doses of cetirizine and fexofenadine may be initiating a compensatory vasodilatory pathway through nitric oxide. It should be emphasized that, in contrast to the effect of mepyramine [16], neither cetirizine nor fexofenadine, even at high doses prevented or reduced atherosclerosis progression in *ApoE^−/−^* mice. It is possible that, in addition to H1-antihistaminic properties, other chemical characteristics unique to these H1-antihistamines may play a role in the proatherogenic effects.

## Supporting Information

Table S1 Primer sequences for genes used in qRT-PCR
(DOC)
