# Supplementary material for: Chronic Ingestion of H1-Antihistamines Increase Progression of Atherosclerosis in Apolipoprotein E-/- Mice
Source: PLoS One. 2014 Jul 14;9(7):e102165. doi: 10.1371/journal.pone.0102165 (PMC4096593; doi:10.1371/journal.pone.0102165)
Supplement: Table S1 — Primer sequences for genes used in qRT-PCR (DOC) [file pone.0102165.s001.doc]

**Table S1.** Primer sequences for genes used in qRT-PCR

| **Genes** | **Primer sequences** |
| --- | --- |
| Beta-actin | Forward 5’- ACGGCCAGGTCATCACTATTG-3’ |
|  | Reverse 5’- CAAGAAGGAAGGCTGGAAAAGA-3’ |
| Egr1 | Forward 5’-TACCCCAAACTGGAGGAGATGA-3’ |
|  | Reverse 5’-GGCAGCACCGAGGAACTG-3’ |
| iNOS | Forward 5’-GGCAGCCTGTGAGACCTTTG-3’ |
|  | Reverse 5’- TGCATTGGAAGTGAAGCGTTT-3’ |
| eNOS | Forward 5’-CTGGCCCAGAAATACCTGGTT-3’ |
|  | Reverse 5’- ACCGAACGAAGTGACACAATCC-3’ |
| COX1 | Forward 5’-GCCAGTGAATCCCTGTTGTTACT-3’ |
|  | Reverse 5’-GGCCGAAGCGGACACA-3’ |
| COX2 | Forward 5’-AGGGTTGCTGGTGGTAGGAA-3’ |
|  | Reverse 5’-GGTCAATGGAAGCCTGTGATACT-3’ |
